# Dermatomyositis as a complication of interferon-α therapy: a case report and review of the literature

**DOI:** 10.1007/s00296-014-2984-4

**Published:** 2014-03-18

**Authors:** Hideyuki Shiba, Tohru Takeuchi, Kentaro Isoda, Yasuhito Kokunai, Yumiko Wada, Shigeki Makino, Toshiaki Hanafusa

**Affiliations:** 1 Department of Internal Medicine (I), Osaka Medical College, Daigaku-Machi 2-7, Takatsuki, Osaka 569-8686 Japan; 2 Department of Dermatology, Osaka Medical College, Takatsuki, Osaka Japan

**Keywords:** Dermatomyositis, Polymyositis, Interferon-α, Hepatitis C, Tacrolimus

## Abstract

Autoimmune disorder is one of the important side effects of interferon-α therapy. Some polymyositis cases as complication of interferon-α therapy were reported, but dermatomyositis were rarely. We report a case of dermatomyositis as a complication of interferon-α therapy for hepatitis C. A 52-year-old Japanese man was treated by combination therapy with pegylated interferon-α-2b and ribavirin for hepatitis C. Three months after the initiation of therapy, he showed erythema in the posterior cervical to dorsal and anterior cervical to thoracic regions, weight loss, general malaise, muscle pain, and severe increase in levels of muscle enzymes. We made a diagnosis of dermatomyositis according to these clinical features, proximal muscle-predominant myogenic change on electromyography, and infiltration of monocytes and CD4+-dominant lymphocytes on skin biopsy, although myositis-associated antibodies were absent. He was successfully treated with intravenous immunoglobulin and tacrolimus in addition to glucocorticoid. This is a very rare case of dermatomyositis associated with interferon-α therapy. We reviewed several similar published cases and the association of dermatomyositis and type I interferon.

## Introduction

Polymyositis (PM) and dermatomyositis (DM) are autoimmune muscle disorders that symmetrically affect primarily the proximal limbs, neck, and pharyngeal muscles. DM is accompanied by a characteristic rash such as Gottron’s papules and heliotrope rash of the eyelids. CD4+ T cells, CD8+ T cells, macrophages, and dendritic cells infiltrate around muscular tissue and blood vessels with degeneration and regeneration of muscular fibers. Inflammatory cytokines such as TNF-α, IL-1, IL-6, IL-15, and IL-18 are thought to play a crucial role in the pathogenesis of PM/DM [1].

Type I interferon (IFN) is a giant cytokine family including IFN-α, IFN-β, IFN-ω, IFN-λ, and IFN-τ. These cytokines have antiviral and antitumor activities. IFN-α and IFN-β are used to treat viral hepatitis or malignant melanoma. Furthermore, the efficacy of IFN-β for multiple sclerosis was also demonstrated [2]. However, several studies reported that autoimmune diseases such as systemic lupus erythematosus and PM/DM can develop during treatment with IFN-α or IFN-β [3–14]. We report a patient who developed DM as a complication of IFN-α therapy for hepatitis C. In this patient, dermatomyositis was rapidly progressive and successfully treated with glucocorticoid therapy in addition to intravenous immunoglobulin (IVIG) and tacrolimus without reactivation of the hepatitis C virus.

## Case report

**Fig. 1:**
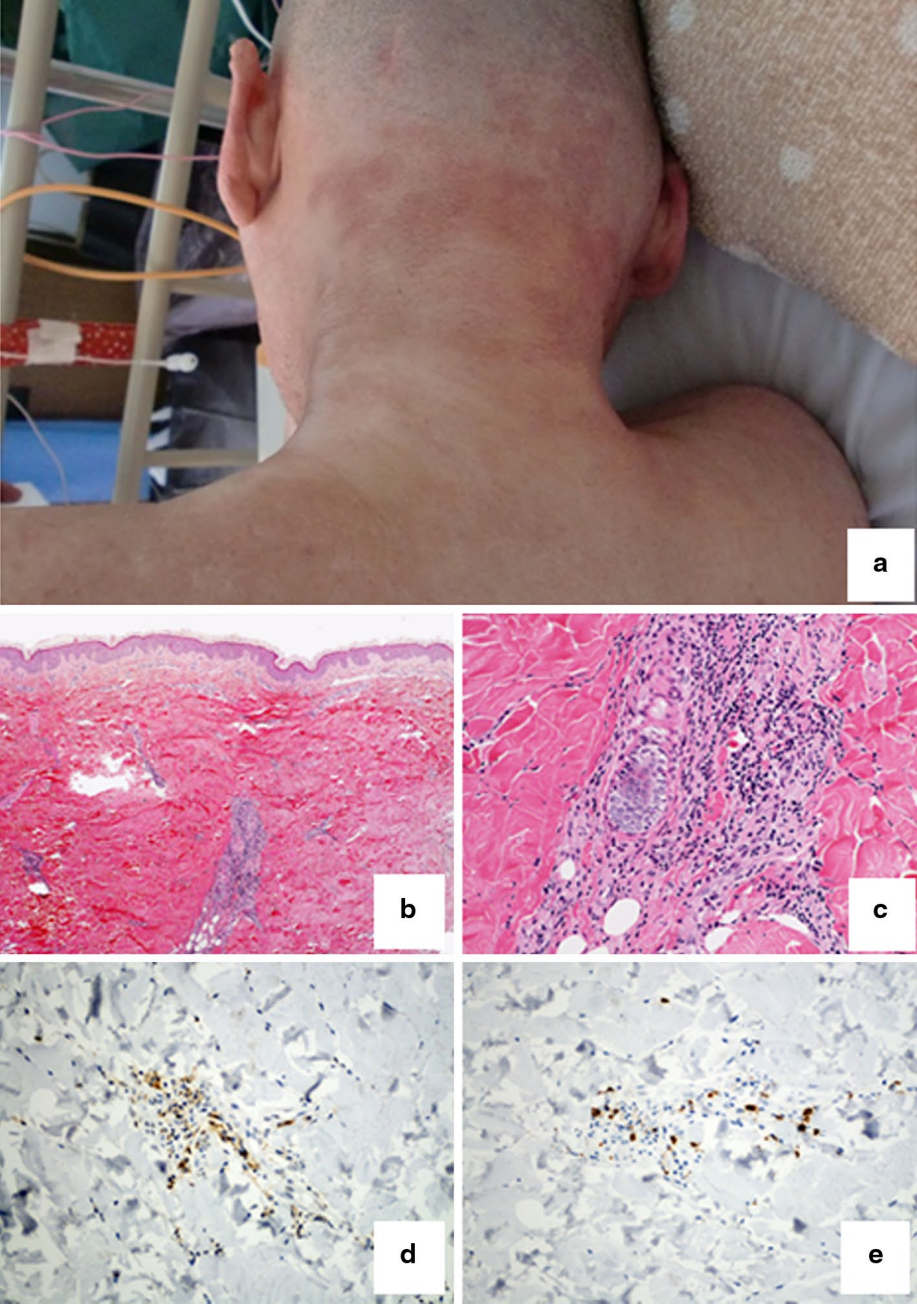
**a** Erythema on the posterior cervical region. **b**, **c** Monocytes and lymphocytes infiltrate around blood vessels in the superficial corium layer and in the middle to deep layers of the corium. (**b** H&E, ×40) (**c** H&E, ×400) **d** and **e** Lymphocytes stained with CD4 infiltrate into the perivascular region more than those with CD8. (**d** immunostaining for CD4, ×400) (**e** immunostaining for CD8, ×400)

A 52-year-old man was diagnosed as being a hepatitis C virus (HCV, Genotype 1) carrier in 1997, but he was not treated. He sought medical advice in regard to hepatitis at a hospital. Combination therapy with weekly subcutaneous injection of pegylated IFN-α-2b (PEG-IFN-α-2b) 100 mg/body and oral administration of ribavirin 800 mg/day were initiated in June 2011. Erythema involving the cervical and dorsal regions appeared, and weight loss was noted 3 months after the initiation of therapy. In January 2012, exacerbation of the erythema, general malaise, muscular pain, and a severe increase in the level of creatine kinase (CK) 23–36,500 U/l were observed. Therefore, PEG-IFN-α-2b and ribavirin were discontinued, and the patient was admitted to our hospital. Heliotrope rash on the eyelids, shawl sign, and protruding and purple-red erythema (Fig. 1) were present on the posterior cervical to dorsal and anterior cervical to thoracic regions. Proximal upper limb-dominant muscular weakness of the cervical flexors and deltoid muscle and decreased grip strength were also observed. The blood examination showed increased levels of CK (29,333 U/l), aldolase (288.8 U/l), C-reactive protein (3.94 mg/dl), aspartate aminotransferase (1,106 U/l), alanine aminotransferase (1,171 U/l), and lactate dehydrogenase (1,709 U/l). The thyroid function was normal. Positive levels of antinuclear antibody (1:640, speckled pattern) were detected. Anti-aminoacyl tRNA synthetase (ARS), anti-signal recognition particle, anti-Mi-2 antibody, anti-PM/SCL antibody, anti-MDA5 antibody, anti-ribonucleoprotein antibody, anti-double stranded DNA antibody, and other disease-specific autoantibodies were negative. Complement level was normal. Electromyography showed proximal muscle-predominant myogenic changes. Magnetic resonance imaging of upper limbs showed diffuse myopathy. A skin biopsy showed infiltration of monocytes and CD4+-dominant lymphocytes around blood vessels in the superficial corium layer and in the middle to deep layers of the corium (Fig. 1). Although a muscle biopsy was not performed because of the patient’s non-consent, based on these findings, a diagnosis of DM was made [3, 4], and prednisolone 80 mg/day and IVIG (400 mg/kg/day × 5 days) were initiated. Because a sufficient decrease in the levels of muscle enzymes was not achieved, administration of tacrolimus (3 mg/day) was initiated 13 days after admission, and IVIG was performed twice more. The serum levels of muscle enzymes decreased to the reference range without the reactivation of HCV.

## Discussion

We experienced a patient who developed DM during IFN-α therapy for hepatitis C. Eleven patients with PM/DM (8 with PM and 3 with DM) related to IFN-α therapy, including our patient, have been reported (Table 1) [5–14]. PM/DM related to IFN-α therapy is more frequent in males. The diseases for which IFN-α therapy was indicated in these patients included hepatitis C in 6 patients, malignant melanoma in 2, hepatitis B in 1, essential thrombocytosis in 1, and chronic myelocytic leukemia in 1. Most patients, including our patient, developed PM/DM within 3 months after the start of IFN-α therapy that ranged from 2 weeks to 7 months in duration. Two previously reported patients with DM were positive for anti-ARS antibodies, such as anti-Jo-1 and anti-PL-7 antibodies, and interstitial pneumonia was concomitantly present [5, 6]. However, these findings were absent in our patient. The PM/DM related to IFN-α in these patients responded to treatment well with a favorable prognosis. In one patient, the discontinuation of IFN-α improved DM spontaneously without immunosuppressive treatment.

**Table 1:** DM and PM associated with IFN alpha therapy

Ref	DM or PM	Age/sex	Disease	IFN	Latency	Autoantibodies	IP	Treatment	Outcome
[3]	DM	62/F	Hepatitis C	PEG-IFN-α-2b	2 weeks	Anti-PL-7 Ab, ANA	+	PDN, IVIG	Improved
[4]	DM	57/F	Malignant melanoma	IFN-α	6 weeks	Anti-Jo-1 Ab	+	DEX, MTX	Improved
[5]	PM	50/F	Hepatitis C	PEG-IFN-α	2 months	None	ND	PDN	Improved
[6]	PM	54/M	Hepatitis C	IFN-α-2b	3–4 months	Anti-thyroglobulin Ab, Anti-microsome Ab	ND	None	Improved
[7]	PM	47/M	Hepatitis C	IFN-α	2 months	ANA	ND	PDN, steroid pulse	Improved
[8]	PM	51/M	Hepatitis C	IFN-α-2b	6 months	ND	ND	ND	Improved
[9]	PM	33/M	Hepatitis B	IFN-α	6 weeks	None	ND	PDN, IVIG	Improved
[10]	PM	50/F	Essential thrombocytosis	IFN-α-2b	2 months	None	ND	PDN, IVIG	Improved
[11]	PM	48/F	Malignant melanoma	IFN-α	7 months	Anti-thyroglobulin Ab, Anti-peroxidase Ab	ND	PDN, steroid pulse	Improved
[12]	PM	24/M	Chronic myeloid leukemia	IFN-α	ND	None	ND	MPDN	Improved
Ours	DM	52/M	Hepatitis C	PEG-IFN-α-2b	3 months	ANA	–	PDN, IVIG, TAC	Improved

*Ref* reference, *DM* dermatomyositis, *PM* polymyositis, *M* male, *F* female, *PEG* pegylated, *IFN* interferon, *Ab* antibodies, *ANA* anti-nuclear antibodies, *PDN* prednisolone, *MPDN* methylprednisolone, *DEX* dexamethasone, *MTX* methotrexate, *TAC* tacrolimus, *IVIG* intravenous immunogloblin, *ND* not described, *IP* interstitial pneumonia

Re-administration of IFN-α should be considered cautiously. It was reported that re-administration of IFN-α caused rapid recurrence of myositis in a patient with inclusion body myositis related to IFN-α [17]. However, the discontinuation of IFN therapy in HCV carriers or the use of steroids in HCV-infected patients may increase the RNA levels of HCV, causing hepatitis [18]. We could find no reports of reactivation of HCV or onset of hepatitis caused by discontinuation of IFN-α or start of immunosuppressive therapy in IFN-α-related DM/PM patients with HCV infection. Reactivation of HCV in our patient was not observed despite strong immunosuppressive therapy with high-dose glucocorticoid and tacrolimus. However, therapeutic strategies for the reactivation of HCV or progression of hepatitis are not established, and care must be taken to avoid these risks.

Type I IFN is involved in the pathogenesis of autoimmune diseases such as systemic lupus erythematosus and PM/DM [19–21]. A large number of plasmacytoid dendritic cells infiltrate into the muscle tissue of patients with PM/DM, and mRNAs of the type I IFN-associated gene (IFN signature) are highly expressed [22]. Myxovirus-resistant protein A, a gene that is induced by type I IFN, is highly expressed in the muscle fibers and blood vessels of these patients [22, 23]. PM/DM as a complication of IFN-α, including that in the present patient, indicates that type I IFN may be involved in the onset of PM/DM, although the exact relation between type I IFN and the development of PM/DM as well as the potential mechanism of disease development remain unclear. These issues must be examined more thoroughly in the future.

We presented a patient who developed DM during the administration of IFN-α for treatment of hepatitis C. He was successfully treated with IVIG and tacrolimus in addition to glucocorticoid despite serious muscular symptoms. Several similar cases of PM/DM as a complication related to IFN-α have been reported. These cases suggest that IFN-α may contribute to the pathogenesis of PM/DM.
